# Predialysis nephrology care amongst Palestinian hemodialysis patients and its impact on initial vascular access type

**DOI:** 10.1080/0886022X.2020.1727512

**Published:** 2020-02-18

**Authors:** Anwar S. Atieh, Ala O. Shamasneh, Abdurrahman Hamadah, Kamel A. Gharaibeh

**Affiliations:** a Department of Internal Medicine, Faculty of Medicine, Al-Quds University, Abu Dis, Palestine; b Department of Internal Medicine, Faculty of Medicine, Hashemite University, Zarqa, Jordan

**Keywords:** End-stage renal disease (ESRD), hemodialysis (HD), arteriovenous fistula (AVF), central venous catheter (CVC), tunneled hemodialysis catheter (TDC), predialysis nephrology care

## Abstract

Referral time for end-stage renal disease (ESRD) patients to nephrologists and initial vascular access method are considered significant factors that impact health outcomes at the time of hemodialysis (HD) initiation. Native arteriovenous fistula (AVF) is strongly recommended as initial access. However, little is known about the referral rate among ESRD receiving HD in Palestine and its correlation with AVF creation. In Ramallah Hemodialysis Center, we investigated the pre-dialysis nephrology care and AVF usage in 156 patients. Type of access at HD initiation was temporary central venous catheter (CVC) in 114 (73%), tunneled hemodialysis catheter (TDC) in 21 (13%) and AVF in 21 (13%). Out of all participants, 120 (77%) were seen by nephrologist prior to dialysis. Of the participants who initiated dialysis with a CVC, 36 (31%) had not received prior nephrology care. All participants who initiated dialysis with functional AVF had received prior nephrology care. Patients who were not seen by a nephrologist prior to HD initiation had no chance at starting HD with AVF, whereas 17% of those who had nephrology care >12 months started with AVF. In conclusion, a relatively large percentage of Palestinian HD patients who were maintained on HD did not have any predialysis nephrology care. In addition, patients who received predialysis nephrology care were significantly more likely to start their HD through AVF whereas all those without predialysis nephrology care started through CVC. More in-depth national studies focusing on improving nephrology referral in ESRD patients are needed to increase AVF utilization.

## Introduction

Early nephrology referral is important for patients with progressive chronic kidney disease (CKD) who are approaching kidney failure [1–3]. Several studies have demonstrated that pre-dialysis nephrology care is associated with better survival rates and lower frequency of hospital admissions [1,4], as it can give nephrologists time for optimal management of multiple care domains including interventions for dialysis planning and preparation in venous access construction once anticipating the start of hemodialysis (HD) [5]. Adequate vascular access is one of the major challenges that a nephrologist faces when HD has been selected as the treatment for end-stage renal failure [4].

Among all available vascular access options, it is generally agreed that native AVF is regarded as the best access for most HD patients and is preferred over AVG and CVC [6–9], due to its longevity, lower mortality risk, and fewer associated complications, among other advantages [4,10–15]. Nonetheless, the number of patients starting HD therapy using a CVC or AVG is still high [16].

In the United States in 2003, The Fistula First Breakthrough Initiative (FFBI) was a project initiated in collaboration with the Centers for Medicare & Medicaid Services (CMS), the End Stage Renal Disease (ESRD) Networks, and the entire renal community as the National Vascular Access Improvement Initiative (NVAII) [7]. One of the primary goals of this national project was to increase the use of AVFs for HD patients. In 2009, the percentage of AVF use exceeded the goal that was set initially [17]. However, recently, because of the very high rates of patients utilizing central venous catheter (CVC) at initial HD in the United States, which nearly exceeding 80% [16,18], ‘a new direction of the FFBI has focused on strategies to reduce CVC use, and subsequently, the FFBI has now been renamed the “Fistula First-Catheter Last Initiative”’ [18].

Unlike other dialysis-related outcomes, attaining optimal vascular access and timely creation of AVF are a complicated process that ideally managed within a multidisciplinary approach and taking into account several factors, including: hospital system, collaboration among providers and issues related to patient preferences and perception [19,20]. Facing this challenge and so increasing the native AVF utilization is facilitated with early referral to the nephrologist in the pre-dialysis period for access planning [1–3,6].

In 2016, according to the U.S. Office of Disease Prevention and Health Promotion’s Healthy People 2020 initiative, the percentage of CKD patients receiving nephrology care from at least 12 months before the start of renal replacement therapy was 36.8%, exceeded the HP2020 goal of 30.0% [21]. Meanwhile, the overall proportion of prevalent arteriovenous fistula (AVF) utilization was 64.1%, and this appears to have plateaued since 2012 [21]. It has set an objective to ‘increase the proportion of chronic kidney disease patients receiving care from a nephrologist at least 12 months before the start of renal replacement therapy’ and ‘increase the proportion of adult HD patients who use AVFs or have a maturing fistula as the primary mode of vascular access at the start of renal replacement therapy’ [21].

In West Bank/Palestine, there are 12 kidney dialysis units [22]. According to the 2017 annual health report of the Palestinian Ministry of Health, the number of HD patients is growing. In this report, a total of 147,494 HD sessions took place in 2016. The overall number of dialysis patients in the West Bank/Palestine has increased from 1014 patients in 2015 to 1119 patients in 2016 [22,23].

Although some studies elaborate important aspects pertaining to the dialysis population in Palestine, to our knowledge, no studies have assessed the pre-dialysis nephrology care and its effect on native AVF utilization in Palestine [24–27]. Hence forward, we conducted this present analysis to evaluate the rate of pre-dialysis nephrology care in Palestinian HD patients and to determine its impact on vascular access type at dialysis initiation. This information is to be used so that measures can be taken to improve fistula creation and timely placement of permanent AV access can be practiced. In addition, it will allow further studies that are needed to look into factors as to why HD patients in Palestine, even when advised AV access, did not undergo dialysis through it.

## Materials and methods

### Study design and setting

In this cross-sectional study, we investigate the rate of pre-dialysis nephrology care and referral to a specialty clinic in Palestinian HD patients and its impact on the type of initial HD access. We recruited all adult participants aged 18–85 years, receiving HD as outpatients from August to December of 2018 at Palestinian Medical Complex Hospital in Ramallah, Palestine which is considered one of the largest Ministry of Health dialysis units in Palestine as per the total number of patients who undergo HD weekly.

### Participants

We screened 198 Palestinian participants who had the diagnosis of ESRD, undergoing regularly scheduled HD sessions of Saturday–Monday–Wednesday or Sunday–Thursday–Tuesday. Exclusion criteria included pediatric age group (less than 18 years), acute dialysis; major mental or neurological illness precludes their ability to be recruited with fully consenting; refused to participate; died before completing their data or those who were unavailable at the time of the study. This study was carried out in accordance with the recommendations of the Al-Quds University Research Ethics Committee with written informed consent from all subjects. The protocol was approved by the Al-Quds University Research Ethics Committee.

### Data collection

All participants underwent in-person interviews either before, after, or during the HD session using structured questions. For each participant, detailed questions regarding nephrology care prior to dialysis initiation, time of first nephrology contact as it relates to the time of initiation dialysis and initial vascular access of dialysis.

Patients’ medical records were all reviewed to collect their demographics and characteristics information. Demographic data collected included age, sex, weight, height, body mass index (BMI). Presence of comorbidities including diabetes mellitus, hypertension, dyslipidemia, coronary artery disease, or cerebrovascular disease was recorded.

*Data pertaining to the cause of ESRD*: Diabetes mellitus, hypertension, polycystic kidney disease, glomerulonephritis, other, or unknown were also obtained.

Regarding to the type of access used for first HD and the current, including temporary CVC, tunneled hemodialysis catheter (TDC), AVF, or AVG. In addition, information was also gathered if patients were aware of their renal disease and followed by nephrologists, which was defined as at least one outpatient visit in the three months prior to commencing HD by a nephrologist. Timely advice for vascular access was considered if patients were informed that they will be needing dialysis therapy soon, and should have permanent vascular access placed prior to starting dialysis therapy.

### Statistical methods

Data were summarized by calculating means and standard deviation (SD) or medians and range for quantitative variables and percentages for categorical variables. Effect of early nephrology referral was assessed at different time points (starting <3 months, 3–12 months, and >12 month prior to dialysis initiation). Categorical variables of interest (impact of early nephrology care vs. no prior care before dialysis, on type of vascular access at initiation) were compared using chi-square test. Descriptive terms were used where appropriate.

## Results

A total of 198 patients were undergoing regular HD at the designated unit during the study period. Of these, 156 patients were enrolled in the study. Forty-two patients were excluded from the study (22 died before completing their data, two declined to participate, three pediatric patients (below age of 18 years old), 15 patients were not available/hospitalized at the time of the study).

Participants’ age ranged from 18 to 85 years (*M* = 55; SD = 15), 92 were males (59%) and 64 were females (41%). Average BMI was (*M* = 26; SD = 6). Twenty-nine (19%) were smokers. The cause of ESRD was diabetes mellitus in 68 (44%), hypertension in 23 (15%), polycystic kidney disease in eight (5%), glomerulonephritis in 21 (13%), other in 19 (12%), and unknown in 17 (11%). Major associated comorbidities were diabetes mellitus in 87 (56%), hypertension in 108 (69%), dyslipidemia in 60 (38%), coronary artery disease in 67 (43%), cerebrovascular disease in 11 (7%), and peripheral vascular disease in 34 (22%). At the time of the study, patients had an average time since starting dialysis of 24 ranged from 1 to 216 months. Details of demographics are presented in Table 1. Type of method of HD access at initiation of dialysis was CVC in 135 (86%) (of the 135, those with temporary catheter-non-tunneled were 114 (73% of the overall group) and those with permanent-tunneled were 21 (13% of the overall group)), AVF in 21 (13%). The average wait time between nephrology referral and fistula creation in months was 2 months and with a range of 0–36 months.

**Table 1.** Baseline demographics and characteristics of study participants.

Patient characteristics	Overall (*n* = 156)
Baseline demographics	
Age (years), mean ± SD	55 ± 15
Gender	
Male, n (%)	92 (59)
Female, n (%)	64 (41)
Weight (kg), mean ± SD	74 ± 16.6
Height (m), mean ± SD	1.66 ± 8.5
BMI (kg/m^2^), mean ± SD	26 ± 6
Smoker, n (%)	29(19)
Cause of ESRD	
Diabetes mellitus, n (%)	68 (44)
Hypertension, n (%)	23 (15)
Adult polycystic kidney disease, n (%)	8 (5)
Glomerulonephritis, n (%)	21 (13)
Other, n (%)	19 (12)
Unknown, n (%)	17 (11)
Associated comorbidities	
Diabetes mellitus, n (%)	87 (56)
Hypertension, n (%)	108 (69)
Dyslipidemia, n (%)	60 (38)
Coronary artery disease, n (%)	67 (43)
Cerebrovascular disease, n (%)	11 (7)
Peripheral vascular disease, n (%)	34 (22)
Time in months since HD initiation, median (range)	24 (1–216)

BMI: body mass index; ESRD: end stage renal disease; HD: hemodialysis; CVC: central venous catheter; AVF: arteriovenous fistula; AVG: arteriovenous graft.

Of the overall group, 120 (77%) were seen by a nephrologist prior to needing dialysis and 36 (23%) reported not having nephrology care prior to dialysis. Length of time from first nephrology contact to HD initiation in those followed in nephrology clinics was 3.9 years (SD = 5.4). Of the ones who were followed in nephrology clinics, one subjects (1%) had first nephrology evaluation in the 3 months prior to starting dialysis, and 25 (21%) had nephrology evaluation in the 12 months prior to starting dialysis. The rest (95 patients, 79%) of those receiving nephrology care prior to dialysis initiation were followed by nephrology starting more than one year prior to HD initiation (Table 2). In the overall study group, of the patients who initiated dialysis with a CVC (*N* = 135), 99 (73%) had received prior nephrology care and 36 (26%) received no prior nephrology care. Of the patients who initiated dialysis with an AVF (*N* = 21), 21 (100%) had received prior nephrology care. Of the 120 patients who received nephrology care prior to dialysis initiation, 21 patients (17.5%) had started dialysis with an AVF whereas in patient without prior nephrology care, none of the patients started with an AVF (*p*=.007) (Table 3).

**Table 2.** Hemodialysis access characteristics.

Hemodialysis access characteristics	Overall (*n* = 156)
Initial HD access method	
Temporary CVC, n (%)	114 (73)
TDC, n (%)	21 (13)
AVF, n (%)	21 (13)
Wait time between nephrology referral and fistula creation, months, median (range)	2 (0–36)
Seen by nephrologist prior to needing dialysis	
Yes, n (%)	120 (77)
No, n (%)	36(23)
Years from nephrology contact to HD initiation in those followed by nephrology, mean (SD)	3.9 (5.4)
First nephrology evaluation was within three months prior to starting dialysis?^a^	
Yes, n (%)	1 (1)
No, n (%)	119 (99)
First nephrology evaluation started more than 12 months prior to starting dialysis?^a^	
Yes, n (%)	95 (79)
No, n (%)	25 (21)

BMI: body mass index; ESRD: end stage renal disease; CVC: central venous catheter; TDC: tunneled hemodialysis catheter; AVF: arteriovenous fistula; AVG: arteriovenous graft; HD: hemodialysis.

^a^ % is from the total of 120 who had nephrology follow up prior to dialysis initiation.

**Table 3.** Initial access characteristics according to pre-dialysis follow up.

Characteristics of initial access	Patients who initiated dialysis through AVF (*N* = 21)	Patients who initiated dialysis through CVC (*N* = 135)
Received pre-dialysis nephrology care (*N* = 120)	21	99
No pre-dialysis nephrology care (*N* = 36)	0	36

CVC: central venous catheter; AVF: arteriovenous fistula.

Of the 95 patients who had nephrology care longer than one year prior to HD initiation, 16 patients (17%) had initiated HD through AVF. Of the 24 patients who had dialysis care within one year but not within 3 months of HD initiation, five (20%) started HD with an AVF. In patients who had initiated HD with an AVF (*N* = 21), no one had nephrology care within 3 months of HD, five had nephrology care within 12 months of dialysis, and 16 patients (76%) had received nephrology care longer than one year (Table 4).

**Table 4.** Initial access characteristics according to duration of pre-dialysis nephrology care.

Characteristics of initial access	Patients who initiated dialysis through AVF (*N* = 21)	Patients who initiated dialysis through CVC among those who had prior nephrology care (*N* = 99)
First nephrology evaluation in the 3 months prior to starting dialysis (*N* = 1)	0	1
First nephrology evaluation in the 12 months prior to starting dialysis (*N* = 24)	5	19
First nephrology evaluation >12 month prior to starting dialysis (*N* = 95)	16	79

CVC: central venous catheter; AVF: arteriovenous fistula.

## Discussion

To the best of our knowledge, this is the first study to investigate the rate of pre-dialysis nephrology care among HD patients in Palestine and its impact on the type of initial vascular HD access. For advanced CKD patients, nephrology referral of all individuals with GFR <30 mL/min/1.73 m^2^, is recommended by Kidney Disease Improving Global Outcomes (KDIGO) 2012 guidelines and stressing that timely nephrology referral maximizes decision making regarding planning for kidney replacement therapy (KRT) to optimize outcomes [28–30].

Our results show that 77% of all participants had pre-dialysis nephrology care and 79% of them (60% of all participants) had nephrology evaluation for more than 12 months prior to starting dialysis. It is evident from previous studies that only a small subgroup of ESRD patients who maintained on HD received prior nephrology care which is usually late, where the late referral was defined as patients who were referred to nephrologists for less than one year prior to dialysis initiation. Our results are in agreement with findings of a Korean prospective cohort study that found among 1088 HD patients, 62.3% were referred early (more than one year prior to dialysis initiation) [31]. This trend is similar to that reported from Europe where it is estimated that 25% of patients are referred very late [32]. In a large cohort study of 443,761 incident ESRD patients in the USA between 2006 and 2010, 33% of recent ESRD patients had received no nephrology care prior to initiation dialysis, while only 28% had received care for >12 months [5].

Guidelines from different countries strongly recommend native AVF because it provides the best access for longevity, decreased risk of infection and lowest association with morbidity and mortality [8,33–39]. On the other hand, catheter use is linked to higher rates of infection and could compromise dialysis adequacy [40,41]. Although the consensus favoring fistula as the vascular access of first choice appears to be strong, the degree of international variation in fistula use is considerable. For example, one Dialysis Outcomes and Practice Patterns Study found that CVCs continue to be the initial HD access in 68.3% of the patients. Not having timely nephrology care seems to limit suboptimal initial vascular access [42]. In our study, 86% of participants initiated dialysis through a CVC both tunneled or non-tunneled catheter and the remaining participants using an AVF as the initial access resembled that of a previous study conducted in the United States between 2005 and 2007 [42]. A study of a cohort of 356 HD patients showed that 75% of patients who reported being seen by a nephrologist at least one month before they maintained on HD were more likely than those referred later to use AV as an initial vascular access (39% versus 10%) and 6 months after starting HD (74% versus 56%). Also in this study, patients who were followed by a nephrologist one month prior to initiating HD therapy used a catheter for a median of 202 days compared with 19 days for patients who followed greater than 12 months prior to initiating HD therapy [43]. Another retrospective analysis of 204 patients conducted in the United States found that late referral to a nephrologist (defined as a referral of CKD patients who are in stage 5) substantially decreased the likelihood of permanent vascular access for initiation of dialysis [44]. In our study, we found that participants who received pre-dialysis nephrology care were significantly more likely to start dialysis with an AVF than those who did not receive (17% versus 0%) nephrology care. Furthermore, all participants, whether they received nephrology care more or less than 12 months before they initiated HD had no difference in the rate of AVF usage as initial vascular access for dialysis. These results could be attributed to patient’s refusal of AVF, late referral to surgical evaluation and too long to surgical appointments.

Early nephrology referral has been associated with multiple advantages, including improved outcomes [45–48], financial cost of emergent dialysis, psychosocial preparation and modality choice of initial dialysis [49]. In addition, early referral may be associated with slowing of the progression of ESRD, decreasing the need for and duration of hospital admission, higher albumin, relatively lower phosphorus and parathyroid hormone levels, choice of peritoneal dialysis modality and improved quality of life [31,44,45,50–53]. Data from a previous prospective cohort study that conducted between 2002 and 2006, suggested that the anemia and progression of left ventricular hypertrophy in the late referral patients during HD treatment are associated with poor survival on HD [54].

Our results show that although 77% had predialysis nephrology care, only 13% of received their dialysis through permanent access as initial vascular access for dialysis and the remaining with a temporary catheter (73%). Although the causes of this are unclear, they could be attributed to physician related factors such as lack of education of the importance of early surgical referral or a dedicated effort to ensure fistula creation, or patient related factors, such as physician does refer the patient to surgery but patient refuses for a multitude of reasons such as denial or lack of proper education and so patient ends up starting HD with a CVC. In our current study, the reasons of this finding were not explored, however, these are important areas to investigate in future studies [55,56]. A previous retrospective cohort study that conducted between 2000 and 2001 to evaluate the relationship between predialysis nephrology care and a range of dialysis-related clinical outcomes, especially the prevalence of permanent vascular access (both fistula and graft) and their results suggested that patients who had intense (defined in this study as >6 nephrology visits during 12 months) predialysis nephrology care had more favorable health outcomes and parameters at the time of dialysis initiation as they were more likely to have permanent vascular access, low prevalence of severe anemia, very low eGFR and low percentage of death within two years of dialysis initiation [1]. In our review of literature, little is known about developing countries in this area and this study helps highlight the need for focus on early nephrology referral. Future studies are needed to focus on the understanding of referral patterns, increasing the frequency of predialysis nephrology care visits because it is important for decisions and therapeutic interventions for dialysis planning and preparation [57]. Furthermore, monitoring how referral patterns impact other health outcomes including vascular access survival, quality of life and mortality among developing countries patients would be of great impact.

## Geolocation information

This study conducted at the national dialysis center in Ramallah, West Bank, Palestine, which is considered one of the largest Ministry of Health dialysis units in Palestine as per the total number of patients who undergo HD weekly.

## Conclusions

A relatively large portion of HD patients in a large dialysis unit in Palestine did not have any pre-dialysis nephrology care. Furthermore, we found a high incidence of CVC use which is contrary to all the recommendations and guidelines of Nephrology societies which is a matter of concern and should lead to action. In addition, pre-dialysis care in terms of placement of AV access prior to initiating HD is better in the hand of a nephrologist, even as late as within three months prior to dialysis initiation. Patients who did not receive nephrology care prior to dialysis had no chance of starting dialysis using AV fistula. Unfortunately, we still have a low incidence of AV utilization even in patients who received pre-dialysis care. More in-depth national studies of factors improving early nephrology referral in advanced CKD and effects of healthcare management issues should be initiated. Besides that, providing a conscious effort, campaign and wide educational programs for physician may improve the low rate of AV placement among group who received pre-dialysis care.
